# Neuroprotective Mechanism of BNG-1 against Focal Cerebral Ischemia: A Neuroimaging and Neurotrophin Study

**DOI:** 10.1371/journal.pone.0114909

**Published:** 2014-12-15

**Authors:** Nai-Fang Chi, Ho-Ling Liu, Jen-Tsung Yang, Jr-Rung Lin, Shu-Li Liao, Bo-Han Peng, Yen-Tung Lee, Tsong-Hai Lee

**Affiliations:** 1 Department of Neurology, Shuang Ho Hospital, Taipei Medical University, New Taipei City, Taiwan, and Department of Neurology, School of Medicine, College of Medicine, Taipei Medical University, Taipei, Taiwan; 2 Department of Medical Imaging and Radiological Sciences, Chang Gung University College of Medicine, Taoyuan, Taiwan; 3 Department of Neurosurgery, Chiayi Chang Gung Memorial Hospital, Chiayi, Taiwan, and Department of Neurosurgery, Chang Gung University College of Medicine, Taoyuan, Taiwan; 4 Clinical Informatics and Medical Statistics Research Center, Chang Gung University, Taoyuan, Taiwan; 5 Department of Traditional Chinese Medicine, Taoyuan General Hospital, Ministry of Health and Welfare, Taoyuan, Taiwan; 6 Department of Traditional Chinese Medicine, Chang Gung Memorial Hospital, Linkou Medical Center, Taoyuan, Taiwan; 7 Department of Traditional Chinese Medicine, En Chu Kong Hospital, New Taipei City, Taiwan; 8 Stroke Center and Department of Neurology, Chang Gung Memorial Hospital, Linkou Medical Center, Taoyuan, Taiwan, and Chang Gung University College of Medicine, Taoyuan, Taiwan; School of Pharmacy, Texas Tech University HSC, United States of America

## Abstract

BNG-1 is a herb complex used in traditional Chinese medicine to treat stroke. In this study, we attempted to identify the neuroprotective mechanism of BNG-1 by using neuroimaging and neurotrophin analyses of a stroke animal model. Rats were treated with either saline or BNG-1 for 7 d after 60-min middle cerebral artery occlusion by filament model. The temporal change of magnetic resonance (MR) imaging of brain was studied using a 7 Tesla MR imaging (MRI) system and the temporal expressions of neurotrophin-3 (NT-3), brain-derived neurotrophic factor (BDNF), and nerve growth factor (NGF) in brain were analyzed before operation and at 4 h, 2 d, and 7 d after operation. Compared with the saline group, the BNG-1 group exhibited a smaller infarction volume in the cerebral cortex in T2 image from as early as 4 h to 7 d, less edema in the cortex in diffusion weighted image from 2 to 7 d, earlier reduction of postischemic hyperperfusion in both the cortex and striatum in perfusion image at 4 h, and earlier normalization of the ischemic pattern in the striatum in susceptibility weighted image at 2 d. NT-3 and BDNF levels were higher in the BNG-1 group than the saline group at 7 d. We concluded that the protective effect of BNG-1 against cerebral ischemic injury might act through improving cerebral hemodynamics and recovering neurotrophin generation.

## Introduction

Stroke is a major cause of death worldwide. The prevention of stroke risk factors can decrease the incidence and mortality of stroke. However, most stroke survivors may suffer from irreversible neurological damage that decreases their quality of life. The effects of many neuroprotective agents on acute stroke have been investigated by clinical trials, but unfortunately only thrombolytic therapy administered in a narrow time window improves neurological outcomes after acute ischemic stroke [1], [2].

BNG-1 is a herb complex used in traditional Chinese medicine that has been used in ischemic stroke therapy for hundreds of years in southern China [3]. BNG-1 consists of 8 components: Scutellaria Radix (6%), Angelica Radix (12%) Coptis Rhizome (12%), Glycyrrhiza Radix (12%), Bupleuri Radix (14%), Ginseng Radix (14%), Astragali Radix (14%), and Bambusa Concretio Silicea (16%) [3]. Compounds in these components exhibit neuroprotective properties against cerebral ischemic injury through antiplatelet or antiinflammation action [4]–[13]. BNG-1 is currently undergoing a Phase III double-blind, randomized, placebo-controlled, multicenter study to compare the functional outcomes and safety of treatment with BNG-1-aspirin with those of aspirin treatment in ischemic stroke recovery (ClinicalTrials.gov identifier: NCT01675115).

A pharmacodynamic study revealed that BNG-1 is a phosphodiesterase (PDE) inhibitor, which exhibited predominant PDE1 inhibition as well as weak PDE3 and PDE6 inhibition [3]. PDE1 regulates vascular contractile function and vascular remodeling [14]. However, few *in* *vivo* PDE1 functional studies have been published, particularly in the ischemic stroke field. It has been proposed that PDE1 inhibitor increases neurotrophin expression through the phosphorylation of cyclic adenosine monophosphate response element-binding protein (CREB) [15]. Therefore, we hypothesized that BNG-1 is beneficial in restoring cerebral hemodynamics and neurotrophin levels after cerebral ischemia. This study was intended to identify the temporal change in cerebral hemodynamics by using magnetic resonance (MR) diffusion/perfusion imaging and neurotrophin protein/mRNA levels in rats treated with BNG-1 after transient focal cerebral ischemia.

## Materials and Methods

### Animal

The animal experimental protocol was approved by the Institutional Animal Care and Use Committee of Chang Gung University and Chang Gung Memorial Hospital, Taiwan. Two groups of male Sprague-Dawley rats were used for the longitudinal MR imaging (MRI) study; one group was treated with BNG-1 and the other was treated with saline (n = 8 in each group). To assess the temporal changes in neurotrophin protein and mRNA, more groups of male rats were used to form a sham operation group (n = 8), and groups that were tested 4 h, 2 d, and 7 d after middle cerebral artery (MCA) occlusion (n = 8 for each time point for the BNG-1 and saline groups). The rats were 12 to 16 wk old and their body weight was 250 to 320 g.

### Middle cerebral artery occlusion model

Transient left MCA occlusion for 60 min was used as an acute ischemic model according to our previous method [16]. Before the surgical procedure, rats were quickly anesthetized with 3% isoflurane in a mixture of air with nitrous oxide and oxygen (69%/30%) and then 1.5% isoflurane was maintained during the experiments. The proximal portion of the external carotid artery (ECA) was tightly ligated with a silk suture. A 20-mm 4-0 nylon surgical thread was inserted from the left ECA into the internal carotid artery (ICA) to occlude the MCA. The left common carotid artery (CCA) was then permanently ligated and the wound was temporarily closed. Anesthesia was discontinued after these procedures were complete. After a 60-min occlusion of the left MCA, the rat was anesthetized again, and the wound was opened to remove the nylon surgical thread for the reperfusion of the MCA. In the sham operation group, similar procedures were conducted without ligation or occlusion of any vessel. Endovascular suture occlusion of the MCA for 60 min would result in irreversible cerebral ischemic injury in both the cerebral cortex and striatum [17]. Therefore, we only included animals that exhibited right-side weakness with upper-limb dominance and had infarction in both the striatum and cortex for the study. The rats with only striatal infarction detected by T2-weighted image on the second day after MCA occlusion were excluded from the study (n = 11).

During ischemia, rectal temperature was monitored in all the animals and was maintained at approximately 37°C with a heating pad and an overhead lamp. After restoration of blood flow, the animals were allowed to recover at ambient temperatures (21°C to 23°C). All the rats used in this study were disinfected with iodine before surgery. After surgery, the surgical wound was checked for signs of infection every 2 days, and gentamycin was administered if there was evidence of wound infection. In addition, 0.3 to 0.5 mg/100 g of meperidine was injected intramuscularly if any symptoms of pain were observed.

Rats were anesthetized with 3% isoflurane in a mixture of air with nitrous oxide and oxygen (69%/30%) before being euthanized. Rats were quickly decapitated and their brains were removed for further experiments.

### BNG-1

BNG-1 was provided as a dry powder by Braingenesis Biotechnology, Ltd, and was dissolved in saline as a vehicle. The oral administration dose of BNG-1 was 1 g/kg at a volume of 10 ml/kg immediately after removal of the nylon surgical thread from ICA and closure of surgical wound, and then every 24 hours for 7 consecutive days [3]. The control group was operated with the same procedure except treated with saline.

### Immunohistochemistry

Three neurotrophins, neurotrophin-3 (NT-3), brain-derived neurotrophic factor (BDNF), and nerve growth factor (NGF), were immunostained according to the protocol described in [16]. The avidin-biotin-peroxidase method (Vector Labs., Burlingame, CA, USA) was used for immunostaining. Briefly, a cycle of brain specimen was cut at a thickness of 20 µm for 10 consecutive slices, and then at a thickness of 400 µm for one slice. Total 20 cycles covering infarcted brain region of 12 mm in length were collected for immunohistochemistry (20 µm) and western blot/polymerase chain reactions (PCR) (400 µm), respectively. After the fresh frozen sections were fixated in ice-cold acetone, they were incubated for one night at 4°C with the first antibodies and were then reacted for 1 h with the biotinylated second antibodies. We used 3,3′-diaminobenzidine tetrahydrochloride and 0.02% H_2_O_2_ for tissue staining. The average optical intensity of immunoreactivity at lesion and non-lesion cortex was calculated from a total of 20 slices per rat, and eight rats at each time point were included for the analysis.

### Western blot

Western blots of NT-3, BDNF, and NGF were studied according to the method described in [18]. The rat cortex was homogenated and the protein content of the supernatant was estimated with an absorbance of 560 nm by using a bicinchoninic acid protein assay reagent kit (Pierce, Rockford, IL, USA). After electrophoresis and protein transfer to a polyvinylidene fluoride membrane, the membrane was stained with primary and secondary antibodies. After these procedures were complete, the membrane was reacted in a chemiluminescence reagent (Western Lightning^TM^, Chemiluminescence Reagent Plus, PerkinElmer Life Sciences, Inc., MA, USA). It was then exposed to a high-performance chemiluminescence film (Amersham Biosciences, England, UK) to measure the optical intensity.

A fluorescence imaging system (Vilber Lourmat, France) was used to quantify the immunoreactivity and western blots according to the method described in [19]. The background optical intensity of the area near the examined cortical area and the immunoreactive band was measured. The optical intensity ratio of the ischemic cortex to that of the sham operation was determined after subtracting the background intensity. The means ± SDs of these values were calculated and were statistically compared at each time point between the 2 groups. Eight rats were included at each time point for the analysis.

### TaqMan real-time reverse-transcription polymerase chain reaction

Real time reverse-transcription (RT) polymerase chain reactions (PCRs) for NT-3, BDNF, and NGF were conducted according to the protocol described in [19]. RNA was isolated from the cortex of each rat after mincing with TRIzol reagent (Life Technologies, NY, USA). Chloroform was used to extract RNA, and the RNA was purified twice by using RNeasy minicolumns. RT-PCR performed using a TaqMan Universal PCR Master Mix kit (Applied Biosystems, USA) and an ABI Prism 7900 Sequence Detection System (Applied Biosystems, USA) was used to measure transcript encoding for NT-3, BDNF, and NGF. 18S rRNA transcript was used as internal control gene and was amplified in a separate tube to normalize for variance in input RNA. The 2(−Delta Delta C(T)) relative quantification method was used to measure the level of mRNA [20]. The mRNA ratio of the ischemic cortex to that of the sham operation rats was determined. The means ± SDs of these ratios were calculated and were statistically compared at each time point between the 2 groups. Eight rats were included at each time point for the analysis.

### Magnetic resonance imaging method

Inhalation of a nitrous oxide/oxygen/isoflurane mixture (69%/30%/1.5%) was used to anesthetize the rats before MRI. After exposing the inguinal area, a biomedical silicone catheter (external diameter of 0.64 mm) was cannulated into the left femoral vein to administer the MR contrast agent. During an MR scan, the rats were placed in the prone position with head fixed in a reproducible position in a nonmagnetic cradle by using an appropriate spacer.

The MR images were obtained using a 7T Clinscan animal MRI system with a bore size of 30 cm (Bruker, Ettlingen, Germany). A volume resonator with a diameter of 72 mm was used for radio frequency transmission and a 4-channel phased array coil optimized for rat brains was used to receive signals. A T2-weighted turbo spin echo sequence was applied to cover most of the brain regions of interest with 20 coronal slices that used the following imaging parameters: TR/TE = 2920 ms/38 ms, ETL = 7, slice thickness = 1 mm, matrix size = 256×256, in-plane resolution = 0.148×0.148 mm^2^. From these images, five slice locations (2 mm thick) were selected based on a bregma of 0.4 mm and an interaural distance of 8.6 mm for T2-weighted imaging with set imaging parameters used for diffusion-tensor imaging (DTI) and dynamic susceptibility contrast (DSC) MRI. DTI used a spin-echo echo-planar imaging (EPI) sequence (TR/TE = 3000 ms/34 ms, matrix size = 128×128, in-plane resolution = 0.3×0.3 mm^2^, averages = 3) with diffusion gradients applied in 30 directions (b = 600 and 1200 mm/s^2^). Parallel imaging was applied using generalized autocalibrating partially parallel acquisitions with an acceleration factor of 2. The apparent diffusion coefficient (ADC) maps were calculated using the DTI images. Perfusion weighted imaging was conducted using a gradient-echo EPI sequence (TR/TE/FA = 600 ms/10 ms/50°, matrix size = 128×128, in-plane resolution = 0.3×0.3 mm, acceleration factor = 2). An amount of 0.3 mL MR contrast media (0.15 mmol dimeglumine gadopentetate contrast; Magnevist, Bayer Schering Pharma, Berlin, Germany) was administered in 3 s at the seventh measurement. DSC-MRI data were processed using nICE software (Nordic ICE, NordicNeuroLab, Bergen, Norway). The arterial input function was semi-automatically obtained from voxels around right MCA (contralateral to the occlusion side) in each animal, based on criteria including peak height and rising time of the concentration time curve. Relative CBF (relCBF) maps were then generated using a SVD deconvolution method [21]. Susceptibility weighted imaging (SWI) was performed between the DWI and DSC scans by using a flow-compensated 3-dimensional gradient-echo sequence with the following parameters: TR/TE/FA = 33 ms/18 ms/40°, matrix size = 512×512, in-plane resolution = 0.078×0.078 mm, average = 2). Thirty-two 0.5-mm-thick coronal slices were obtained from the acquisition slab. Maximum intensity projection (MIP) images were then calculated to generate slices with identical locations and thicknesses as the ADC and CBF maps. Brain MR scans were longitudinally examined in all rats 1 d before and 4 h, 2 d, and 7 d after MCA occlusion.

### Measurement of infarction volume

All the 20 slices of T2-weighted images at each time point (4 h, 2 d, and 7 d) after left MCA occlusion were used for calculation of infarction volume. The area with high signal intensity in cortical and striatal brain regions was identified and measured using Mongo software (Research Imaging Institute, San Antonio, TX, USA). A diffusion weighted images (b = 1000 mm/s^2^) were used to identify ventricle regions filled with cerebrospinal fluid and were substracted from the infarction areas. We collected 20 coronal slices of T2-weighted images (i.e. 1 mm apart) totally covering the brain region of 2.0 cm in length. The total infarction area (mm^2^) of cortex and striatum in each coronal slice of each animal was measured and corrected to exclude the effect of brain edema [corrected infarction area = actual infarction area × (non-lesion hemisphere area/lesion hemisphere area)]. The infarction volume (mm^3^) was then calculated by corrected infarction area (mm^2^)×specific distance (1 mm) in altogether 20 slices and was expressed as mean ± standard deviation (SD) for each rat. As our previous study had shown there was good correlation between histological damage and MR infarction size [22], the MR infarction volume was used to compare statistically between saline and BNG-1 groups.

### Analysis of the parameters on magnetic resonance images

In the ADC, MIP of SWI, and CBF measurements, a template of four predetermined regions of interest at the bilateral parietal cortex and striatum was applied for the spatial transformation analysis based on T2-weighted MR images [23] according to our previous method [24]. The spatial transformation procedure was conducted using SPM5 software (Wellcome Department of Cognitive Neurology, London, UK) implemented in Matlab 7.0 (Mathworks Inc, Sherborn, MA, USA). For the ADC, MIP of SWI, and the CBF map, the mean values obtained in the bilateral parietal cortex and the bilateral striatum calculated from all the pre-operation rat brains were used as a baseline value. The low values on the ADC and MIP images and high values on the CBF images (after subtracting the baseline values) were analyzed for every post-operation brain image. In all 5 slices, the ADC, MIP, and CBF images covering a brain region of 1.4 cm in length were used to identify and measure the area (mm^2^) with ischemia-related changes in the left hemisphere using Mongo software (Research Imaging Institute, San Antonio, TX, USA) at 4 h, 2 d, and 7 d after left MCA occlusion. The volumes (mm^3^) of low signal intensity on the ADC and MIP images and high signal intensity on the CBF images were calculated by area (mm^2^)×specific distance (2 mm) in altogether 5 slices for each rat and were expressed as means ± SDs at each time point. The volume of the ADC, MIP intensity, and CBF change were statistically compared between saline and BNG-1 groups.

### Statistical analysis

The researchers who performed the MCA occlusion model (SL Liao) and the analysis of MR images and neurotrophin expression (YT Lee) were blind to the oral administration of BNG-1 or saline (BH Peng). The immunoreactivity, western blot, and RT-PCR optical intensity ratios as well as the volume of the ADC, MIP, and CBF signal intensity changes in MR images were compared between saline and BNG-1 groups at each time point by Mann-Whitney *U* test. Kruskal-Wallis test (non-parametric one-way ANOVA) with LSD post-hoc test was used to compare each time point to pre-operation value in saline and BNG-1 groups. Statistical significance was defined as *P*<0.05. SPSS (Version 13) software was used to examine the statistics.

## Results

**Figure 1:**
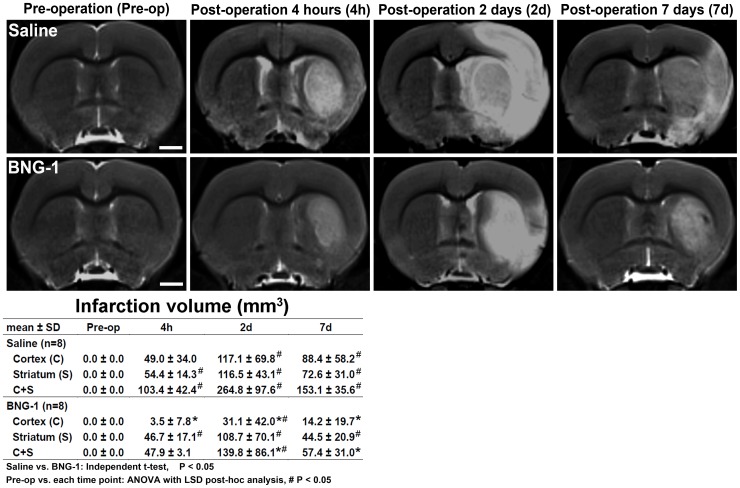
Comparison of the temporal changes in the infarction volumes after focal cerebral ischemia between the saline and BNG-1 treatments. The T2-weighted image indicates that the infarction volume (white region) on the lesion cortex is smaller in the BNG-1 group than the saline group from as early as 4 h to 7 d. No striatum difference was observed. The bar in the pre-operation panels indicates 2.5 mm.

The infarction volume measured using the T2-weighted image is presented in Fig. 1. The BNG-1 group exhibited a significantly lower infarction volume in the lesion hemisphere compared with the saline group, from as early as 4 h to 7 d after MCA occlusion. If the cortex and striatum were compared separately, the infarction volume decreased significantly only in the cortex, and not in the striatum, of the BNG-1 group (BNG-1 vs. saline group in the cortex: 3.5±7.8 vs. 49.0±34.0 mm^3^ at 4 h, *P* = 0.019; 31.1±42.0 vs. 117.1±69.8 mm^3^ at 2 d, *P* = 0.011; 14.2±19.7 vs. 88.4±58.2 mm^3^ at 7 d, *P* = 0.008). However, no significant difference occurred in the striatum at any time point (*P*>0.05). The temporal change of T2-weighted images showed a significant increase of infarction volume from post-operation 4 h to 7 d and most severe at 2 d compared with pre-operation (*P*<0.05) in both groups.

**Figure 2:**
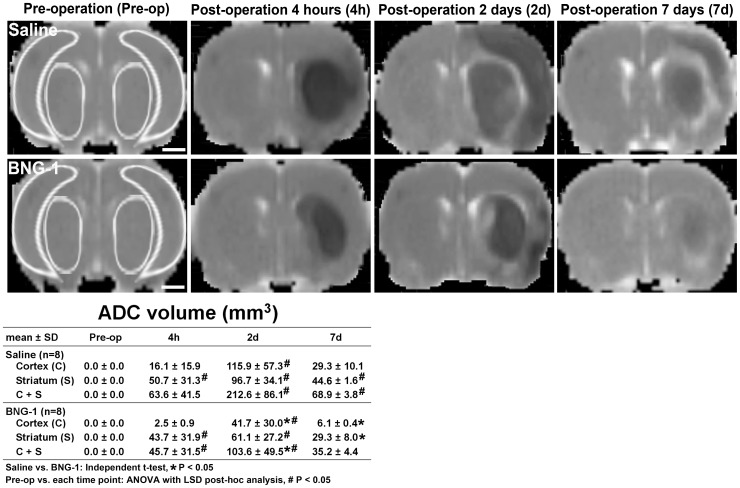
Comparison of the temporal changes in the ADCs after focal cerebral ischemia between the saline and BNG-1 treatments. The ADC indicates that the lesion volume (dark region) is smaller in the BNG-1 group than the saline group at 2 d and 7 d on the lesion cortex and at 7 d on the lesion striatum. The bar in the pre-operation panels indicates 2.5 mm. The template for region of interest is demonstrated in the brain map of pre-operation rat, which is used for quantitative measurement of ADC, CBF and MIP.

The volume analysis of the ADC signal intensity change is presented in Fig. 2. The BNG-1 group exhibited a significantly lower volume of low signal intensity on the lesion cortex compared with the saline group at 2 and 7 days after MCA occlusion (BNG-1 vs. saline group: 41.7±30 vs. 115.9±57.3 mm^3^ at 2 d, *P* = 0.033; 6.1±0.4 vs. 29.3±10.1 mm^3^ at 7 d, *P* = 0.022). At 7 d, the BNG-1 group also reflected a significantly lower volume of low signal intensity on the lesion striatum compared with the saline group (BNG-1 vs. saline group: 29.3±8 vs. 44.6±1.6 mm^3^ at 7 d, *P* = 0.031). The temporal change of ADC images showed a significant increase of low signal intensity volume from post-operation 4 h to 7 d and most severe at 2 d compared with pre-operation (*P*<0.05) in both groups.

**Figure 3:**
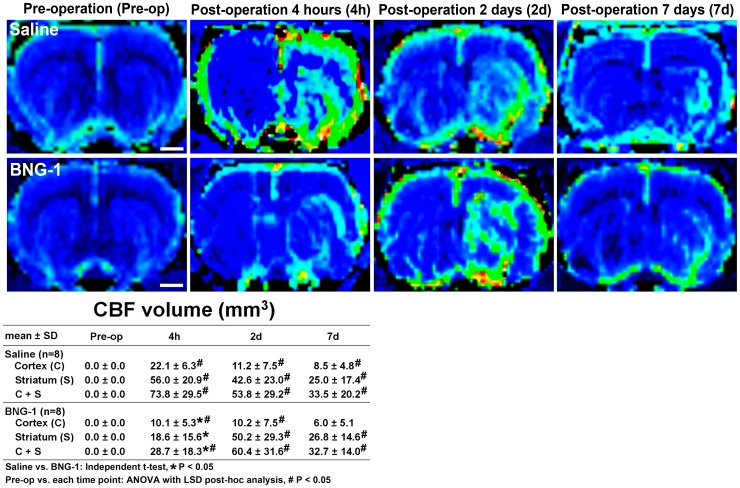
Comparison of the temporal changes in the CBF after focal cerebral ischemia between the saline and BNG-1 treatments. CBF indicates that the postischemic hyperperfusion area (green area) is smaller in the BNG-1 group than the saline group at 4 h in both the lesion cortex and striatum, whereas no difference occurred after 2 d. The normal CBF region is blue. The bar in the pre-operation panels indicates 2.5 mm.

Fig. 3 presents the volume analysis of the CBF signal intensity change. The BNG-1 group exhibited a significantly lower volume of high signal intensity in both lesion cortex and striatum compared with the saline group at 4 h after MCA occlusion (BNG-1 vs. saline group: 10.1±5.3 vs. 22.1±6.3 mm^3^ in the cortex, *P* = 0.028; 18.6±15.6 vs. 56±20.9 mm^3^ in the striatum, *P* = 0.012). However, the 2 groups were not significantly different after the second day (*P*>0.05). The temporal change of CBF images showed a significant increase of high signal intensity volume from post-operation 4 h to 7 d and most severe at 2 d compared with pre-operation (*P*<0.05) in both groups.

**Figure 4:**
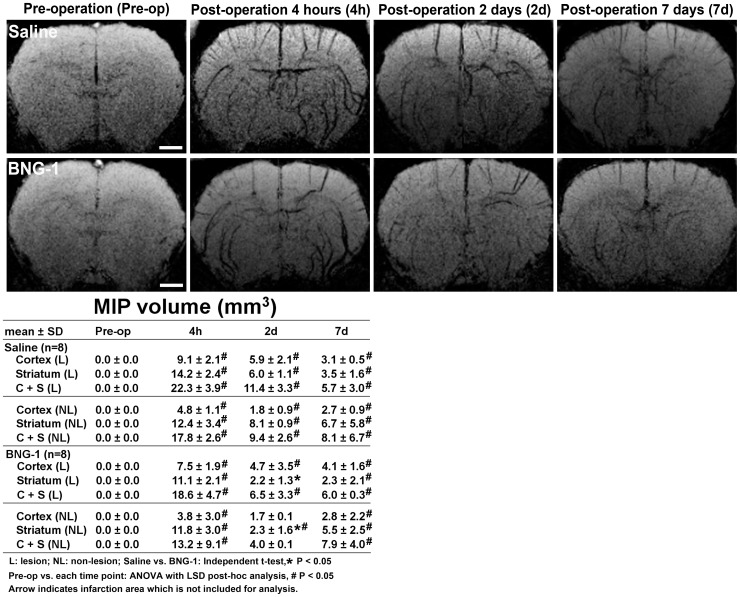
Comparison of the temporal changes in the maximal intensity projection after focal cerebral ischemia between the saline and BNG-1 treatments. The maximal intensity projection of the SWI indicates that the volume of the low signal intensity (cerebral vessels) is smaller in the BNG-1 group than the saline group in the striatum of both lesion and non-lesion hemispheres at 2 d. The bar in the pre-operation panels indicates 2.5 mm.

The volume analysis of the signal intensity change measured using the MIP of SWI is presented in Fig. 4. The BNG-1 group exhibited a significantly lower volume of low signal intensity in the striatum of both lesion and non-lesion hemispheres compared with the saline group at 2 d after MCA occlusion (BNG-1 vs. control group: 2.2±1.3 vs. 6±1.1 mm^3^ on the lesion hemisphere, *P* = 0.046; 2.3±1.6 vs. 8.1±0.9 mm^3^ on the non-lesion hemisphere, *P* = 0.036). However, no significant differences were observed at any other time point on the lesion and non-lesion hemispheres between the BNG-1 and saline groups (*P*>0.05). The temporal change of MIP images showed a significant increase of low signal intensity volume from post-operation 4 h to 7 d and most severe at 4 h compared with pre-operation (*P*<0.05) in bilateral hemispheres of both groups.

**Figure 5:**
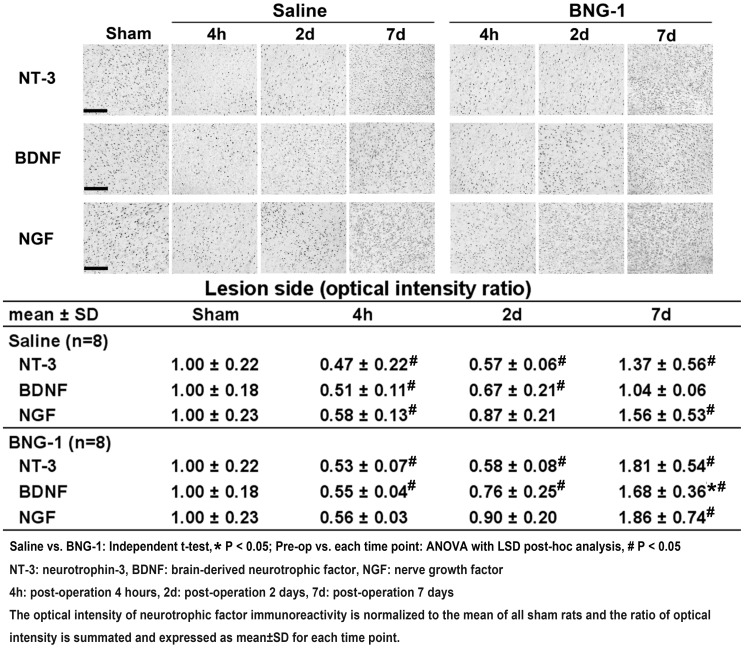
Comparison of the temporal expression of neurotrophic factor immunoreactivity after focal cerebral ischemia between the saline and BNG-1 treatments. The optical intensity of BDNF immunoreactivity on the lesion cortex is higher in the BNG-1 group than the saline group at 7 d, whereas no difference in NT-3 and NGF levels occurred at any time point. The bar in the sham panels indicates 100 µm.

Fig. 5 presents the immunoreactivity results for NT-3, BDNF, and NGF. The optical intensity ratios of NT-3, BDNF, and NGF decreased from post-operation 4 h to 2 d but recovered to above the pre-operation level at 7 d compared with pre-operation (*P*<0.05) in both the BNG-1 and saline groups except the BDNF in saline group (*P*>0.05). There was no significant difference between the two groups at each time point (*P*>0.05). Except at 7 d, BDNF increased significantly more in the BNG-1 group than the saline group (BNG-1 vs. saline group: 1.68±0.36 vs. 1.04±0.06, *P* = 0.040).

**Figure 6:**
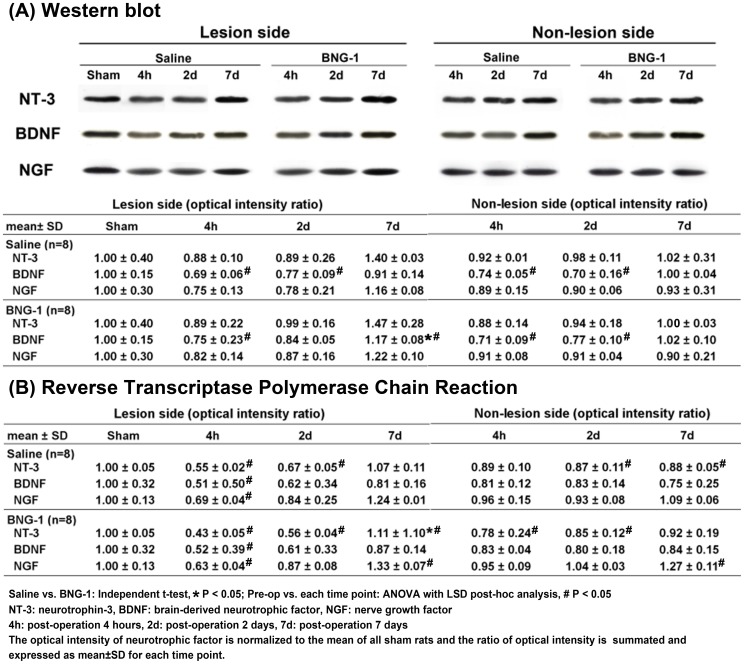
Comparison of the temporal expression of neurotrophic factor protein and mRNA after focal cerebral ischemia between the saline and BNG-1 treatments. **A**: Western blot indicates that the BDNF level on the lesion cortex is significantly higher in the BNG-1 group than the saline group at 7 d, whereas no difference in NT-3 and NGF levels occurred at any time point. **B**: Reverse-transcription PCR indicates that the NT-3 mRNA level on the lesion cortex is significantly higher in the BNG-1 group than the saline group at 7 d, whereas no difference in BDNF and NGF levels occurred at any time point.

The results of the western blots of NT-3, BDNF, and NGF are presented in Fig. 6A. The optical intensity ratios of BDNF, but not NT-3 and NGF, decreased from post-operation 4 h to 2 d on the lesion and non-lesion cortices in both the BNG-1 and saline groups (*P*<0.05). However, BDNF recovered to above pre-operation level at 7 d only in the BNG-1 group (*P*<0.05). There was no significant difference at each time point between the two groups (*P*>0.05). Except, BDNF increased significantly more in the BNG-1 group than the saline group at 7 d on the lesion cortex (BNG-1 vs. saline group: 1.17±0.08 vs. 0.91±0.14, *P* = 0.028).

Fig. 6B presents the results of RT-PCRs of NT-3, BDNF, and NGF. The optical intensity ratios of NT-3, BDNF, and NGF decreased from post-operation 4 h to 2 d mainly on the lesion cortex in both the BNG-1 and saline groups (*P*<0.05), but NT-3 and NGF recovered to above pre-operation level at 7 d only in the BNG-1 group (*P*<0.05). There was no significant difference of NT-3, BDNF and NGF at each time point between the two groups (*P*>0.05). Except, NT-3 increased significantly in the BNG-1 group compared with the saline group at 7 d on the lesion cortex (BNG-1 vs. saline group: 1.11±1.1 vs. 1.07±0.11, *P* = 0.018).

## Discussion

In a previous animal study, a permanent surgical occlusion in an MCA model demonstrated a reduction of the infarction volume in the cortical area after BNG-1 treatment [3]. In the present study, we used a distinct method with transient endovascular suture occlusion in an MCA model [16], which injured both cortical and subcortical areas, and find out that BNG-1 mainly provides a protective effect in the cortical area and less likely in the striatum. This protective effect occurred from as early as 4 h after the first dose of BNG-1 and remained 7 d after MCA occlusion (Fig. 1). These results suggested that the first dose of BNG-1 administered immediately after ischemic injury can induce neuroprotection, and this protective effect can be maintained with continuous post-operation treatment of BNG-1.

A study demonstrated that the PDE1 inhibitor, vinpocetine, increased cerebral perfusion and oxygen extraction in stroke patients [25]. However, a meta-analysis of vinpocetine in acute ischemic stroke did not support its efficacy because of a lack of high-quality study designs [26]. BNG-1 is a PDE1 inhibitor [3]. It is likely that the neuroprotective mechanism of BNG-1 might act as a PDE1 inhibitor against cerebral ischemic and reperfusion injury.

This MRI study demonstrated that BNG-1 is effective at reducing the infarction volume (T2 image in Fig. 1) and brain edema (ADC in Fig. 2) after ischemic injury. The superior recovery of the T2 signal after ischemia in the BNG-1 group compared with the saline group suggested an earlier resolution of vasogenic edema [27], [28], which is typically caused by vascular regulatory dysfunction after blood-brain barrier disruption. The lower ADC signal intensity in the BNG-1 group than the saline group also indicated an earlier resolution of cytotoxic edema [27], [29], which is typically caused by cellular metabolic impairment after ischemia.

A delayed increase in the MR signal intensity occurred in both the CBF and cerebral blood volume from Days 1 to 14 in the ipsilateral cortex after transient MCA occlusion in rats [28]. A previous study revealed that postischemic hyperperfusion was neither correlated to the final infarction nor did it affect the outcome [30], whereas another study reported that postischemic hyperperfusion was associated with an increased infarction volume [29]. Similar findings have been reported and were attributed to increased angiogenesis [28] and vascular regulatory dysfunction after ischemia [31]. Our study revealed that after ischemia, the CBF increase on the lesion hemisphere was lower in the BNG-1 group than the saline group, suggesting an improved postischemic hyperperfusion injury after BNG-1 treatment.

MR SWI is a type of blood-oxygenation-level-dependent imaging, which is correlated with the oxygen consumption of brain tissue [32]. Cerebral vessels filled with deoxyhemoglobin typically present with low signal intensity. Prominent cerebral vessel patterns in SWI can appear in cerebral ischemia, reflecting increased deoxyhemoglobin in vessels. Therefore, the prominent vessel pattern area in SWI is typically a surrogate of a misery perfusion area in acute ischemic stroke. In patients with acute ischemic stroke, the prominent cerebral vessel pattern in SWI is typically alleviated by reperfusion therapy [33]. The BNG-1 group normalized the MIP of SWI signal intensity earlier than the saline group did at 2 d after ischemia, suggesting an earlier normalization of deoxyhemoglobin levels after BNG-1 treatment.

In the central nervous system, neurotrophic factors are associated with neurite outgrowth and cell survival. The highest levels of NT-3, BDNF, and NGF are observed in the hippocampus and cerebral cortex [34]. PDE1 inhibition might increase the phosphorylation of CREB, which increases the transcription of BDNF [15]. Four of the 8 components of BNG-1 (Angelicae, Bupleuri, Ginseng, and Astragali) effectively increased neurotrophic factors in a neurological disease model [35]–[38]. In our study, the BDNF protein and NT-3 mRNA levels of the BNG-1 group were significantly higher than those of the saline group at 7 d after ischemia. It is possible that the neuroprotective mechanism of BNG-1 is partially caused by its effect on recovering neurotrophin levels for neurogenesis after ischemia.

The reason why there is effect only in the cortex but not in the striatum is likely due to the protective effect mainly on neuronal cells and less on fiber tract. Another possible explanation is the better collateral flow from leptomeningeal arteries to cortex than striatum, which results in better survival of cortex tissue than striatal one as well as better absorption of neuroprotective agents. Similar pattern of cortex-dominant protective effect has been reported in some neuroprotective agents such as minocycline and risperidone [39], [40].

Our study had limitations. The relatively heterogeneous results among the animals might have affected the statistical power. Because BNG-1 is a mixed compound with multiple effects on the physiology of stroke, it is difficult to focus on a single therapeutic mechanism against ischemic injury.

In conclusion, this study indicated that BNG-1 treatment can improve cerebral hemodynamics before the recovery of neurotrophin levels. The protective mechanism of BNG-1 against ischemic injury might act by alleviating brain edema, reducing postischemic hyperperfusion injury, normalizing deoxyhemoglobin levels, and recovering neurotrophin generation.
